# A single-center retrospective study of hospitalized COVID-19 patients: demographics, laboratory markers, neurological complications, ICU admission, and mortality

**DOI:** 10.1097/MS9.0000000000000949

**Published:** 2023-06-12

**Authors:** Maria A. Garcia-Dominguez, Bahadar S. Srichawla, Peter Pacut, Jared Quast, Shravan Sivakumar, Jillian Belgrad, Ashwin Panda, Sara Carbone, Delia T. Sanders, Eli Min, Nicole T. Hayes, Abigail Bose, Vanessa Lee, Vincent Kipkorir, Mehdi Ghasemi

**Affiliations:** a University of Massachusetts Chan Medical School Worcester, Massachusetts, USA; b Department of Human Anatomy and Physiology, University of Nairobi, Nairobi, Kenya

**Keywords:** coronavirus disease 2019, intensive care unit, neurological manifestation, mortality, severe acute respiratory syndrome coronavirus-2

## Abstract

The coronavirus disease 2019 (COVID-19) pandemic has unveiled a wide array of clinical biomarkers, and neurological manifestations in affected patients, necessitating further exploration.

**Methods:**

This single-center retrospective study evaluated clinical and neurological sequelae, demographics, as well as laboratory markers, in hospitalized COVID-19 patients from January to September 2020.

**Results:**

Among 1248 inpatients (median age: 68 years; 651 women), 387 (31%) were admitted to the ICU. Central nervous system (CNS) manifestations were present in 521 (41.74%) patients, while peripheral nervous system manifestations were observed in 84 (6.73%). COVID-19-related mortality occurred in 314 (25.16%) cases. ICU-admitted patients were predominantly male (*P*<0.0001), older (age≥60; *P*=0.037) and had more comorbidities such as diabetes (*P*=0.001), hyperlipidemia (*P*=0.043), and coronary artery disease (*P*=0.015). ICU patients exhibited more CNS manifestations (*P*=0.001), including impaired consciousness (*P*<0.0001) and acute cerebrovascular disease (*P*=0.023). Biomarkers linked to admission to the ICU included elevated white blood cell count, ferritin, lactate dehydrogenase, creatine kinase, blood urea nitrogen, creatinine, and acute phase reactants (e.g. erythrocyte sedimentation rate and C-reactive protein). ICU patients demonstrated lower lymphocyte and platelet counts compared to non-ICU patients. Those with CNS involvement in the ICU often exhibited elevated blood urea nitrogen, creatinine, and creatine kinase levels. Higher mortality from COVID-19 was observed in ICU patients (*P*<0.0001).

**Conclusions:**

Multiple serum biomarkers, comorbidities, and neurological manifestations in COVID-19 patients have been consistently documented and may be linked to increased morbidity, ICU admission, and mortality. Recognizing and addressing these clinical and laboratory markers is essential for effective COVID-19 management.

## Introduction

Highlights

- Patients with central nervous system involvement were more likely to require ICU admission and had a higher mortality rate due to coronavirus disease 2019 (COVID-19).
- Elevated white blood cell count, ferritin, lactate dehydrogenase, creatine kinase, blood urea nitrogen, creatinine, and acute phase reactants were identified as biomarkers associated with ICU admission in COVID-19 patients.
- ICU-admitted patients were more likely to have comorbidities, including diabetes, hyperlipidemia, and coronary artery disease.
- Patients admitted to the ICU were more likely to be male and older than 60 years.
- More than 40% of patients had central nervous system manifestations, indicating that neurological signs and symptoms are common in COVID-19 patients and may be associated with increased morbidity and mortality.

Manifestations of severe acute respiratory syndrome coronavirus-2 (SARS-CoV-2) often include the respiratory system; however, reports of neurological manifestations have been implicated early on during the first few outbreaks of the virus (i.e. reports from Madrid in 2020)^1–5^. A wide spectrum of neurological manifestations involves the central nervous system [(CNS), e.g. transverse myelitis, encephalitis, seizure, etc.] and peripheral nervous system [(PNS), i.e. Guillain–Barre syndrome, peripheral neuropathy, etc.), as well as skeletal tissue (i.e. rhabdomyolysis)^6,7^. The pathophysiology of these various disease processes across the neural axis are not well known but may be secondary to a hyperimmune response (i.e. cytokine storm)^8^. Despite this hypothesis, cases of isolated neurological manifestations without primary pulmonary or systemic disease have been reported^9^. These neurological manifestations have been reported in a wide spectrum of ages from adolescence to the elderly^10^. SARS-CoV-2 is an emerging viral pathogen and the causative agent of a worldwide pandemic not seen since the influenza outbreak of 1919. In the present study, we aim to provide a robust analysis on the varying demographics, systemic, and neurological manifestation of those admitted secondary to COVID-19. We stratify our findings based on those requiring ICU admission. Furthermore, a breakdown of comprehensive laboratory findings including acute phase reactants is conducted based on localization to the CNS, PNS, and skeletal muscle. We aim to provide clinicians guidance in identifying high risk COVID-19 patient populations requiring ICU level of care using patient demographics, symptomatology, and biomarkers.

## Methods

### Study design and patients

In this single-center retrospective observational study, we assessed admitted patients from January 2020 to September 2020 who tested positive for SARS-CoV-2 in PCR via nasopharyngeal swab. Patients without a positive PCR test for SARS-CoV-2 were excluded. Furthermore, patients who tested positive but were not admitted to the hospital were also removed from this study. Further assessment including patient demographics, risk factors, ancillary and laboratory tests upon admission as well as neurological manifestations stratified by localization to the CNS and/or PNS. The first reported case of COVID-19 in Massachusetts, USA occurred in March 2020. The peak month of hospitalizations and death secondary to COVID-19 within the study period was in April 2020. This study was conducted under the supervision and approval by the local institutional review board. This work has been reported in line with Strengthening the reporting of cohort, cross-sectional and case-control studies in surgery (STROCSS) criteria^11^. The local institutional review board granted a Health Insurance Portability and Accountability waiver to complete this study.

### Data collection

Convenience sampling was utilized for data collection. For each patient, we reviewed demographic characteristics including age, BMI, sex, and ethnicity. The medical history of all patients was assessed and comorbidities such as hypertension (established diagnosis, use of antihypertensive medications, or systolic blood pressure of≥140 mm Hg or diastolic blood pressure of≥90 mmHg on two separate occasions in the out-patient setting), diabetes mellitus (defined according to the National Diabetes Data Group and WHO), hyperlipidemia, chronic kidney disease, pulmonary disease (includes asthma, chronic obstructive pulmonary disease, and interstitial lung disease), prior cerebrovascular disease, malignancy, and associated risk factors such as smoking were reported. Systemic symptoms including fever, cough, anorexia, diarrhea, throat pain, and abdominal pain were also extracted. Neurological signs and symptoms were retrospectively reviewed and grouped by localization to the CNS, PNS, and/or skeletal muscle injury. Although neurodiagnostic data (i.e. MRI of the brain and spinal cord, electroencephalogram [EEG], electromyography/nerve conduction studies, cerebrospinal fluid (CSF) studies, etc.) were not reported in this study, they were reviewed alongside with the patients’ medical records for further assessment of neurological manifestations upon admission. Localization was determined retrospectively by a single board-certified neurologist, which includes CNS manifestations (dizziness/lightheadedness, headache, impaired consciousness, acute cerebrovascular disease, ataxia, movement disease, seizure, and myelopathy), PNS manifestations (impaired smell, impaired vision, and neuropathy or nerve pain), and skeletal muscle injury. Any documented change of consciousness level (somnolence, stupor, and coma) or content (confusion and delirium) was considered as impaired consciousness in this study. Acute cerebrovascular disease includes ischemic or hemorrhagic stroke diagnosed by documented clinical symptoms, physical examination, and brain imaging including head/neck CT or MR angiography (CTA or MRA), and brain MRI. Seizure was based on the clinical symptoms at the time of presentation alone or combined with the results of EEG upon admission. Skeletal muscle injury was defined as the presence of myalgia plus elevated creatine kinase (CK) greater than 200 U/L^12^. Patients were stratified to those requiring ICU level of care versus non-ICU level of inpatient care. Criteria for ICU admission included single or multiorgan failure necessitating intensive care. All cases requiring ICU admission were reviewed by a board-certified medical or neurointensivist.

Laboratory data was collected upon admission. Furthermore, we grouped laboratory data by both ICU status as well as the presence of CNS symptoms, PNS symptoms, or skeletal muscle injury. Laboratory tests reviewed included a complete blood count, a comprehensive metabolic panel, inflammatory markers (i.e. erythrocyte sedimentation rate, C-reactive protein [CRP], and D-dimer), coagulation markers (i.e. prothrombin time, partial thromboplastin time, and international normalized ratio), CK, ferritin, and lactate dehydrogenase (LDH).

### Statistical analysis

All statistical analyses were performed using SAS statistical software (version 9.4, SAS Institute). The normality of the data was examined using the Shapiro–Wilk test. Normally distributed continuous variables were reported as mean and SD, whereas not normally distributed variables were reported as median and range. Categorical variables were expressed as counts and percentages. Continuous variables were compared by using the Wilcoxon rank sum test with the provision of related Z scores in tables. Proportions for categorical variables were compared using the χ^2^ test. Moreover, the relative risk (RR, with related 95% CI) for ICU admission (as an outcome measure) for each of the categorical variables were calculated. The significance threshold was set as a *P* value less than 0.05.

## Results

### Demographic and clinical characteristics

A total of 1248 patients were included in this retrospective study. Table 1 provides a summary of the clinical and demographic data grouped by those admitted to the ICU (387 cases, 31%) and those who were not (861 cases, 69%). The median age on presentation was 68 years [range, 7–102 year; 69 year in ICU (range, 26–96) and 67 years in non-ICU-admitted patients (range, 7–102)], and 651 (52.16%) individuals were males. Men more often needed ICU admission for management of COVID-19 [239 (61.76%) vs. 412 (47.85%), *P*<0.0001]. Compared with women, there was a 1.48-fold higher ICU admission in men with COVID-19 [relative risk (RR) 1.48, 95% CI 1.25–1.76). Patients admitted to the ICU over the age of 60 were more prevalent [275 (71.06%) vs. 560 (65.04%), *P*=0.037]. The ICU admission in these patients were 1.21 times of those at or less than 60 years old (RR 1.21, 95% CI 1.01–1.46). The most common ethnicity recorded was Caucasian (66.27%). Overall, COVID-19 patients with at least one comorbidity had 1.47 times higher ICU admission compared with patients with no comorbidities (RR 1.47, 95 CI% 1.13–1.91; *P*=0.002) comorbidities than the non-ICU group. This includes diabetes mellitus [161 (41.60%) vs. 274 (31.82%); RR 1.33, 95% CI 1.13–1.57; *P*=0.001], hyperlipidemia [197 (50.90%) vs. 385 (44.71%); RR 1.19, 95% CI 1.01–1.40; *P*=0.043], coronary artery disease [85 (21.96%) vs. 140 (16.26%); RR 1.28, 95% CI 1.06–1.55; *P*=0.015] and heart failure [77 (19.90) vs. 107 (12.44%); RR 1.44, 95% CI 1.18–1.74; *P*=0.001]. Fever (oral temperature>100.4 ^o^ *F*) was more prevalent in the ICU versus non-ICU-admitted patients [240 (62.01%) vs. 462 (53.66%); RR 1.27, 95% CI 1.07–1.51; *P*=0.006].

**Table 1:** Clinical characteristics of inpatients with COVID-19.

	*N* (%)					
Characteristic	Total (*N*=1248)	ICU-Admitted (*N*=387)	Not ICU-Admitted (*N*=861)	RR^a^	95% CI	χ^2^ test *P* ^b^
Age≥60 years	835 (66.91)	275 (71.06)	560 (65.04)	1.21	1.01–1.46	**0.037**
Sex, Male	651 (52.16)	239 (61.76)	412 (47.85)	1.48	1.25–1.76	**<0.0001**
Race						0.119
White	827 (66.27)	247 (63.82)	580 (67.36)	0.90	0.76–1.07	0.221
African American	110 (8.81)	43 (11.11)	67 (7.79)	1.29	1.01–1.66	0.055
Hispanic or Latino	197 (15.78)	58 (14.99)	139 (16.14)	0.94	0.74–1.19	0.604
Asian	44 (3.52)	19 (4.91)	25 (2.90)	1.41	0.99–2.00	0.076
Other	70 (5.61)	20 (5.17)	50 (5.81)	0.92	0.63–1.34	0.650
Comorbidities	1033 (82.77)	339 (87.6)	694 (80.6)	1.47	1.13–1.91	**0.002**
Hypertension	766 (61.38)	238 (61.5)	528 (61.32)	1.01	0.85–1.19	0.953
Diabetes mellitus	435 (34.86)	161 (41.60)	274 (31.82)	1.33	1.13–1.57	**0.001**
Hyperlipidemia	582 (46.63)	197 (50.90)	385 (44.71)	1.19	1.01–1.40	**0.043**
Coronary Artery Disease	225 (18.03)	85 (21.96)	140 (16.26)	1.28	1.06–1.55	**0.015**
Lung Disease^c^	313 (25.08)	109 (28.16)	204 (23.70)	1.17	0.98–1.40	0.092
Heart Failure	184 (14.74)	77 (19.90)	107 (12.44)	1.44	1.18–1.74	**0.001**
Chronic Kidney Disease	257 (20.59)	88 (22.74)	169 (19.63)	1.14	0.93–1.38	0.209
Cerebrovascular disease	193 (15.46)	69 (17.83)	124 (14.43)	1.18	0.96–1.46	0.121
Malignancy	121 (9.70)	41 (10.59)	80 (9.29)	1.10	0.85–1.44	0.472
Current smoker	106 (8.49)	38 (9.82)	68 (7.89)	1.17	0.90–1.54	0.260
General (typical) symptoms						
Fever	702 (56.25)	240 (62.01)	462 (53.66)	1.27	1.07–1.51	**0.006**
Cough	604 (48.40)	195 (50.39)	409 (47.50)	1.08	0.92–1.28	0.346
Anorexia	320 (25.64)	93 (24.03)	227 (26.36)	0.92	0.75–1.11	0.382
Diarrhea	184 (14.74)	49 (12.66)	135 (15.68)	0.84	0.65–1.08	0.164
Throat pain	77 (6.17)	24 (6.20)	53 (6.15)	1.01	0.71–1.42	0.975
Abdominal pain	105 (8.41)	26 (6.72)	79 (9.18)	0.78	0.56–1.11	0.148
Nervous system symptoms						
CNS, any	521 (41.74)	188 (48.58)	333 (38.67)	1.32	1.12–1.55	**0.001**
Dizziness/lightheadedness	61 (4.89)	15 (3.88)	46 (5.34)	0.78	0.50–1.23	0.266
Headache	129 (10.34)	38 (9.57)	91 (10.57)	0.94	0.71–1.25	0.687
Impaired consciousness	336 (26.92)	143 (36.95)	193 (22.42)	1.59	1.35–1.87	**<0.0001**
Acute cerebrovascular disease	18 (1.44)	10 (2.58)	8 (0.93)	1.81	1.19–2.76	**0.023**
Ataxia	34 (2.72)	13 (3.36)	21 (2.44)	1.24	0.80–1.92	0.356
Movement disease	24 (1.92)	9 (2.33)	15 (1.74)	1.21	0.72–2.05	0.488
Seizure	22 (1.76)	6 (1.55)	16 (1.86)	0.88	0.44–1.74	0.702
Myelopathy	19 (1.52)	5 (1.29)	14 (1.63)	0.85	0.40–1.80	0.656
PNS, any	84 (6.73)	25 (6.46)	59 (6.85)	0.96	0.68–1.34	0.798
Impaired smell	38 (3.04)	8 (2.07)	30 (3.48)	0.67	0.36–1.25	0.178
Impaired vision	14 (1.12)	3 (0.78)	11 (1.28)	0.69	0.25–1.88	0.436
Neuropathy or nerve pain	34 (2.72)	14 (3.62)	20 (2.32)	1.34	0.89–2.02	0.194
Myalgia	208 (16.67)	54 (13.95)	154 (17.89)	0.81	0.63–1.04	0.085
Skeletal muscle injury^d^	30 (10.27)	17 (13.28)	13 (7.93)	1.34	0.95–1.88	0.135
Death due to COVID-19	314 (25.16)	188 (48.58)	126 (14.63)	N/A	N/A	**<0.0001**

a RR, relative risk for ICU admission.

b *P* values indicate differences between ICU-admitted and not ICU-admitted in patients with COVID-19 using χ^2^ test, and *P* less than 0.05 was considered statistically significant.

c Lung disease includes asthma, chronic obstructive pulmonary disease (COPD), and interstitial lung disease (ILD).

d Skeletal muscle injury is defined as when a patient had skeletal muscle pain (myalgia) and elevated serum creatinine kinase (CK) level greater than 200 U/L (from total 292 available data). N/A, not applicable.

Bold values signify statistical significance.

Overall, 28 brain MRI and/or MRA [12 (42.86%) in the ICU-admitted patients], three spinal cord MRI [2 (66.67%) in the ICU cases] and 42 head/neck CTA [16 (38.1%) in the ICU cases] were performed in COVID-19 patients upon their admission. Initial presentation with at least one CNS complaint was significantly different among the two groups (RR, 1.32, 95% CI 1.12–1.55; *P*=0.001). From the CNS specific manifestations, impaired consciousness [143 (36.95%) vs. 193 (22.42%); RR 1.59, 95% CI 1.35–1.87, *P*<0.0001] and acute cerebrovascular disease [10 (2.58%) vs. 8 (0.93%); RR 1.81, 95% CI 1.19–2.76; *P*=0.023] were more prevalent in the ICU-admitted group. Based on the results of neuroimaging data (i.e. brain MRI/MRA and head/neck CTA) among the 18 patients with acute cerebrovascular disease, 14 (1.12%) patients had ischemic stroke [7 (0.56%) ICU vs. 7 (0.56%) non-ICU cases] and 4 (0.32%) patients had hemorrhagic stroke [3 (0.24) ICU vs. 1 (0.08%) non-ICU cases]. Clinical seizure was documented in 22 (1.76%) COVID-19 patients upon admission. However, there was no significant difference between ICU [6 (0.48%)] and non-ICU [16 (1.28%)] admitted COVID-19 patients with seizure (RR 0.88, 95% CI 0.44–1.74; *P*=0.702). Among the 22 COVID-19 patients with seizure upon admission, 9 (0.72%) patients underwent EEG, which showed epileptiform discharges, generalized slowing, and normal results in two (both non-ICU case), four (one ICU and three non-ICU cases), and one (non-ICU case) patients, respectively. In the two remaining cases (one ICU and one non-ICU case), EEG only revealed generalized rhythmic delta activity or GRDA. Death due to COVID-19 was also significantly higher in the ICU-admitted patients [188 (48.58%) vs. 126 (14.63%), *P*<0.0001]. CK was only available or checked for 292 patients upon their admission. Accordingly, skeletal muscle injury upon admission was not significantly different between ICU and non-ICU-admitted groups [17 (13.28%) vs. 13 (7.93); RR 1.34, 95% CI 0.95–1.88; *P*=0.135].

### Laboratory findings inpatients without and with admission to the ICU

Table 2 provides a summary of laboratory values among patients with COVID-19 grouped by admission to the ICU and non-ICU group. Data is represented by the median and range (minimum-maximum). Patients requiring ICU admission had a higher white blood cell (WBC) count [7.5 (1.0–31.3) vs. 6.3 (0.9–69.7), *P*<0.0001], absolute neutrophil count [5.97 (0.3–28.5) vs. 4.5 (0.3–83.8), *P*<0.0001]. ICU-admitted patients were more lymphopenic [0.80 (0.1–199.0) vs. 1.0 (0.0–63.4), *P* <0.0001], and thrombocytopenic [194 (25–755) vs. 205 (0.6–737), *P*=0.012]. Acute phase reactants such as erythrocyte sedimentation rate [77 (2–120) vs. 57 (6–120), *P* =0.031], CRP [137.9 (1.2–400) vs. 56.4 (0.07–2200), *P*<0.0001], D-dimer [1.52 (0.19–5000) vs. 1.2 (0.135–5000), *P*=0.005], ferritin [490 (2–7470) vs. 298.15 (0.31–7500), *P*<0.0001] and LDH [371 (7.6–4706) vs. 269 (1.13–1644), *P*<0.001] were significantly elevated in ICU-admitted patients. Moreover, ICU-admitted patients had higher levels of serum CK [207 (14–60 000) vs. 86 (2–60 000), *P*<0.0001] and aspartate aminotransferase [40 (8–10 750) vs. 31 (3–1193), *P*<0.0001]. Both elevated blood urea nitrogen [(BUN), 25 (0.9–141) vs. 18 (0.59–171), *P*<0.0001] and creatinine [1.12 (0.2–18) vs. 0.95 (0.28–52), *P*<0.0001] were also present in the ICU-admitted patients compared to non-ICU patients. Elevated coagulation markers, including prothrombin time [11.1 (9.3–79) vs. 10.8 (1.43–96.5), *P*=0.008] and partial thromboplastin time [33.8 (20.7–150) vs. 31.3 (0.9–139), *P*<0.0001] were observed inpatients admitted to the ICU.

**Table 2:** Laboratory findings of inpatients with COVID-19

	Median (range)				
Characteristic [*N* of Available or Checked Data]	Total (*N*=1248)	ICU-Admitted (*N*=387)	Not ICU-Admitted (*N*=861)	Wilcoxon test Z score	*P* ^a^
Count, ×10^9^/l,					
White blood cell [1220]	6.55 (0.9–69.7)	7.5 (1.0–31.3)	6.3 (0.9–69.7)	5.18	**<0.0001**
Neutrophil [1190]	4.9 (0.3–83.8)	5.97 (0.3–28.5)	4.5 (0.3–83.8)	6.50	**<0.0001**
Lymphocyte [1189]	0.9 (0.0–199.0)	0.80 (0.1–199.0)	1.0 (0.0–63.4)	−6.71	**<0.0001**
Platelet [1215]	201 (0.6–755)	194 (25–755)	205 (0.6–737)	−2.26	**0.012**
ESR, mm/h [93]	67 (2–120)	77 (2–120)	57 (6–120)	2.16	**0.031**
CRP, mg/L [797]	80.9 (0.7–2200)	137.9 (1.2–400)	56.4 (0.07–2200)	9.41	**<0.0001**
D-dimer, mg/l [659]	1.31 (0.135–5000)	1.52 (0.19–5000)	1.2 (0.135–5000)	2.57	**0.005**
Fibrinogen, mg/dl [118]	487.5 (2–6455)	514.5 (2–860)	476.5 (216–6455)	1.13	0.129
Ferritin, ng/ml [564]	369 (0.31–7500)	490 (2–7470)	298.15 (0.31–7500)	5.67	**<0.0001**
LDH, U/L [465]	296 (1.13–4706)	371 (7.6–4706)	269 (1.13–1644)	7.01	**<0.0001**
CK, U/L [292]	114.5 (2–60 000)	207 (14–60 000)	86 (2–60 000)	5.66	**<0.0001**
Aminotransferase, U/L					
Alanine (ALT) [1075]	24 (2.0–2090)	25 (0.9–141)	24 (1.0–1276)	1.55	0.061
Aspartate (AST) [1076]	34 (3–10 750)	40 (8–10 750)	31 (3–1193)	5.84	**<0.0001**
BUN, mg/dl [1204]	20 (0.59–171)	25 (0. –141)	18 (0.59–171)	8.38	**<0.0001**
Creatinine, mg/dl [1211]	1 (0.2–52)	1.12 (0.2–18)	0.95 (0.28–52)	5.03	**<0.0001**
PT, s [635]	10.9 (1.43–96.5)	11.1 (9.3–79)	10.8 (1.43–96.5)	2.38	**0.008**
PTT, s [384]	32.6 (0.9–150)	33.8 (20.7–150)	31.3 (0.9–139)	4.05	**<0.0001**
INR [637]	1 (0.37–38.4)	1 (0.46–8)	1 (0.37–38.4)	1.70	0.088

a *P* values indicate differences between ICU-admitted and not admitted patients with COVID-19, and *P* less than 0.05 was considered statistically significant using the Wilcoxon Two-Sample Test.

BUN, blood urea nitrogen; CK, creatine kinase; CRP, C-reactive protein; ESR, erythrocyte sedimentation rate; INR, International normalized ratio; LDH, lactate dehydrogenase; PT, prothrombin time; PTT, partial thromboplastin time.

Bold values signify statistical significance.

Of note, CSF studies were performed in a total of four (two ICU vs. two non-ICU-admitted) patients with COVID-19. CSF protein level [183.5 (40–327) vs. 15 (2–28); P=0.123], glucose level [115.5 (48–175) vs. 39 (2–76); P=0.349], and WBC count [64.5 (9–120) vs. 1.5 (1,2); *P*=0.123] were not statistically different between ICU and non-ICU-admitted groups.

### Laboratory findings in patients with and without CNS symptoms

Table 3 represents the laboratory markers inpatients with and without CNS symptoms. Patients were further stratified based on ICU admission status. Data is represented by the median value with a range (minimum-maximum). In the subset of patients presenting with CNS symptoms platelets were significantly lower in the total group [191 (6.4–737) vs. 210 (0.6–755), *P*=0.0007], and non-ICU group [190 (6.4–737) vs. 217 (0.6–660), *P*<0.0001]. CK levels were significantly elevated in both subgroup of ICU (*P*=0.043) and non-ICU (*P*=0.047) admitted patients with CNS symptoms compared to those with no CNS symptoms, it was more prevalent in the total group of patients with CNS symptoms [166 (11–60 000) vs. 97 (2–60 000), *P*=0.0007]. BUN was significantly elevated in the total group of patients with CNS symptoms [23 (0.9–171) vs. 18 (0.59–155), *P*<0.0001], ICU group with CNS symptoms [26 (0.9–141) vs. 23 (1–137), *P*=0. 009] and non-ICU group [21 (3–171) vs. 16 (0.59–155), *P*<0.0001]. Amongst those who had seizures, the most common antiseizure medication utilized was levetiracetam. Treatment for those who were diagnosed with a noninfectious immune mediated myelopathy (e.g. transverse myelitis) were treated with high dose intravenous methylprednisolone for a total of 3–5 days. Headache was most often treated supportively with oral acetaminophen. No specific treatment outside of supportive care was provided with those who developed an acute movement disorder (e.g. parkinsonism).

**Table 3:** Laboratory findings of inpatients with COVID-19 with any central nervous system (CNS) symptoms

	Median (range)											
	Total (*N*=1248)				ICU-Admitted (*N*=387)				Not ICU-Admitted (*N*=861)			
Characteristic [*N* of Available/Checked data in total]	Without CNS symptoms (*N*=727)	With CNS symptoms (*N*=521)	Wilcoxon test Z score	*P* ^a^	Without CNS symptoms (*N*=199)	With CNS symptoms (*N*=188)	Wilcoxon test Z score	*P* ^a^	Without CNS symptoms (*N*=528)	With CNS symptoms (*N*=333)	Wilcoxon test Z score	*P* ^a^
Count, ×10^9^/l												
White blood cell [1220]	6.6 (1.3–69.7)	6.5 (0.9–29.2)	−0.03	0.489	7.3 (1.8–31.3)	7.6 (1–29.2)	1.14	0.128	6.4 (1.3–69.7)	6.2 (0.9–23.3)	−1.63	0.051
Neutrophil [1190]	4.8 (0.6–83.8)	5 (0.3–75.4)	−0.14	0.443	5.8 (0.6–28.5)	6.02 (0.3–26.7)	0.88	0.189	4.6 (0.7–83.8)	4.2 (0.3–75.4)	−1.85	**0.032**
Lymphocyte [1189]	0.9 (0–63.4)	0.9 (0.1–199)	−0.96	0.167	0.7 (0.1–4.4)	0.8 (0.1–199)	0.62	0.267	1 (0–63.4)	1 (0.1–17.8)	−0.88	0.190
Platelet [1215]	210 (0.6–755)	191 (6.4–737)	−3.21	**0.0007**	186.5 (46–755)	196.5 (25–685)	0.25	0.400	217 (0.6–660)	190 (6.4–737)	−3.90	**<0.0001**
ESR, mm/h [93]	61 (2–6455)	73 (4–120)	1.40	0.081	74.5 (2–111)	85 (4–120)	−1.28	0.099	56.5 (6–120)	51 (7–112)	0.28	0.390
CRP, mg/l [797]	82.6 (0.5–2200)	78.1 (0.07–400)	−1.20	0.115	142.5 (1.2–400)	130.3 (2.4–400)	−0.47	0.318	62 (0.5–2200)	45.7 (0.07–400)	−2.51	**0.006**
D-dimer, mg/l [659]	1.31 (0.13–5000)	1.32 (0.19–5000)	−0.85	0.197	1.40 (0.23–5000)	1.6 (0.19–5000)	0.19	0.426	1.3 (0.13–5000)	1.10 (0.19–5000)	−1.50	0.067
Fibrinogen, mg/dl [118]	470 (2–6455)	524 (157–813)	1.22	0.112	480 (2–4980)	530 (157–813)	−0.71	0.238	465 (216–6455)	502 (280–779)	0.67	0.250
Ferritin, ng/ml [564]	354.5 (2–7500)	397.3 (0.31–7470)	1.67	**0.048**	487 (2–4980)	490.6 (33–7470)	0.82	0.206	296 (2–7500)	309.2 (0.31–5529)	0.41	0.341
LDH, U/L [465]	303.5 (1.13–4521)	285 (3.7–4706)	0.53	0.298	343 (7.6–4521)	426 (106–4706)	1.76	**0.039**	278.5 (1.13–1644)	254 (3.7–960)	−1.73	**0.042**
CK, U/L [292]	97 (2–60 000)	166 (11–60 000)	3.19	**0.0007**	156.5 (14–60 000)	285 (19–7977)	−1.72	**0.043**	71 (2–1213)	100 (11–60 000)	1.68	**0.047**
Aminotransferase, U/L												
Alanine (ALT) [1075]	23 (1–2090)	25 (3–397)	0.70	0.240	24 (3–2090)	26 (4–397)	1.66	0.051	23 (1–1276)	24 (3–264)	−0.55	0.293
Aspartate (AST) [1076]	32 (8–10 750)	35 (3–2640)	1.39	0.083	37.5 (11–10 750)	42 (8–2640)	1.61	0.054	31 (8–1193)	31 (3–292)	−0.21	0.417
BUN, mg/dl [1204]	18 (0.59–155)	23 (0.9–171)	5.76	**<0.0001**	23 (1–137)	26 (0.9–141)	2.35	**0.009**	16 (0.59–155)	21 (3–171)	4.75	**<0.0001**
Creatinine, mg/dl [1211]	0.94 (0.28–52)	1.07 (0.2–35)	3.75	**<0.0001**	1.01 (0.32–15.9)	1.22 (0.2–18)	2.11	**0.017**	0.92 (0.28–52)	1 (0.34–35)	2.51	**0.006**
PT, s [635]	10.9 (8.9–96.5)	11 (1.43–79)	1.52	0.064	10.9 (9.3–44.8)	11.1 (9.3–79)	−0.78	0.217	10.8 (8.9–96.5)	10.9 (1.43–53.1)	1.10	0.135
PTT, s [384]	32.3 (0.9–139)	32.8 (10.3–150)	1.07	0.142	33.3 (22.8–139)	35.6 (20.7–150)	−1.27	0.103	31.3 (0.9–139)	31.3 (10.3–112.6)	0.21	0.415
INR [637]	1 (0.37–38.4)	1 (0.46–8)	0.92	0.178	1 (0.9–4.3)	1.1 (0.46–8)	−1.22	0.112	1 (0.37–38.4)	1 (0.9–5.4)	−0.02	0.493

a *P* values indicate differences between inpatients with COVID-19 without versus with CNS symptoms, and *P* less than 0.05 was considered statistically significant using the Wilcoxon Two-Sample Test.

BUN, blood urea nitrogen; CK, creatine kinase; CRP, C-reactive protein; ESR, erythrocyte sedimentation rate; INR, International normalized ratio; LDH, lactate dehydrogenase; PT, prothrombin time; PTT, partial thromboplastin time.

Bold values signify statistical significance.

### Laboratory findings in patients with and without PNS symptoms

Table 4 represents the laboratory markers inpatients with and without PNS symptoms. Data is represented by the median value with range. Patients were further stratified based on ICU admission status. Decreased WBC counts were recorded both in the total number of patients [5.9 (1–17.1) vs. 6.6 (0.9–69.7), *P*=0.047] as well as non-ICU patients with PNS symptoms [5.8 (2.8–17.1) vs. 6.35 (0.9–69.7), *P*=0.044]. Elevated fibrinogen was observed in patients with PNS symptoms admitted to the ICU with COVID-19 [763 (498–860) vs. 481.5 (2–7470), *P*=0.007]. Decreased creatinine was recorded in the total group of patients [0.86 (0.2–7.98) vs. 1.01 (0.28–52), *P*=0.0007], ICU group [0.99 (0.2–3.43) vs. 1.14 (0.32–18), *P*=0.016], and non-ICU group with PNS symptoms [0.85 (0.34–7.98) vs. 0.97 (0.28–52), *P*=0.010].

**Table 4:** Laboratory findings of inpatients with COVID-19 with peripheral nervous system (PNS) symptoms

	Median (range)											
	Total (*N*=1248)				ICU-Admitted (*N*=387)				Not ICU-Admitted (*N*=861)			
Characteristic [N of available/Checked data in total]	Without PNS symptoms (*N* =1164)	With PNS symptoms (*N*=84)	Wilcoxon test Z score	*P* ^a^	Without PNS symptoms (*N*=362)	With PNS symptoms (*N*=25)	Wilcoxon test Z score	*P* ^a^	Without PNS symptoms (*N*=802)	With PNS symptoms (*N*=59)	Wilcoxon test Z score	*P* ^a^
Count, ×10^9^/l												
White blood cell [1220]	6.6 (0.9–69.7)	5.9 (1–17.1)	−1.68	**0.047**	7.5 (1.5–31.3)	7.6 (1–16)	−0.39	0.346	6.35 (0.9–69.7)	5.8 (2.8–17.1)	−1.71	**0.044**
Neutrophil [1190]	4.9 (0.3–83.8)	4.53 (0.7–75.4)	−1.16	0.246	5.94 (0.3–28.5)	6.1 (0.7–14.6)	−0.01	0.497	4.5 (0.3–83.8)	4.16 (1.7–75.4)	−1.35	0.088
Lymphocyte [1189]	0.9 (0–199)	0.9 (0.1–17.8)	0.35	0.362	0.8 (0.1–199)	0.7 (01–2.8)	−1.40	0.081	1 (0–63.4)	1.1 (0.2–17.8)	1.32	0.094
Platelet [1215]	201 (0.6–755)	191 (52–537)	−0.56	0.286	194 (25–755)	170 (52–428)	−1.16	0.122	205 (0.6–737)	206 (56–537)	0.004	0.498
ESR, mm/h [93]	67 (2–120)	45.5 (7–120)	−0.82	0.205	77 (2–120)	N/A	N/A	N/A	55.5 (6–120)	45.5 (7–120)	−0.53	0.299
CRP, mg/l [797]	83.4 (0.07–2200)	54.7 (1–283.9)	−2.11	**0.017**	141 (1.2–400)	82.3 (2.4–283.9)	−2.10	**0.018**	56.5 (0.07–2200)	40.2 (1–277)	−1.10	0.136
D-dimer, mg/l [659]	1.33 (0.13–5000)	1.03 (0.22–2970)	−1.5	0.067	1.52 (0.19–5000)	2.00 (0.3–2670)	0.44	0.331	1.25 (0.13–5000)	0.83 (0.22–2970)	−2.10	**0.018**
Fibrinogen, mg/dl [118]	480 (2–6455)	542 (230–860)	1.4	0.080	481.5 (2–7470)	763 (498–860)	2.48	**0.007**	478 (216–6455)	444 (230–642)	−0.79	0.215
Ferritin, ng/ml [564]	373 (0.31–7500)	334.4 (11–2561)	−1.38	0.083	487 (2–7470)	675.6 (86.4–2561)	0.82	0.208	305.3 (0.31–7500)	196 (11–1561.1)	−1.86	**0.032**
LDH, U/L [465]	296 (1.13–4706)	289 (115–640)	−0.060	0.476	370 (7.6–4706)	374 (167–640)	−0.18	0.430	270 (1.13–1644)	250 (115–629)	−0.19	0.424
CK, U/L [292]	119 (2–60 000)	110 (20–574)	−0.16	0.436	204 (14–60 000)	279 (20–574)	−0.37	0.357	86 (1–60 000)	72.5 (34–543)	0.49	0.314
Aminotransferase, U/L												
Alanine (ALT) [1075]	24 (1–2090)	26 (5–182)	0.61	0.272	25 (3–2090)	23 (5–182)	0.34	0.368	23 (1–1276)	27 (6–152)	0.48	0.315
Aspartate (AST) [1076]	34 (3–10 750)	33 (8–363)	−0.36	0.365	40 (9–141)	40 (8–363)	0.41	0.408	31 (3–1193)	32 (11–172)	−0.11	0.455
BUN, mg/dl [1204]	20 (0.59–171)	16 (4–91)	−3.19	**0.0007**	26 (0.9–141)	18 (6–91)	−1.34	0.090	18 (0.59–171)	14 (4–83)	−2.88	**0.002**
Creatinine, mg/dl [1211]	1.01 (0.28–52)	0.86 (0.2–7.98)	−3.18	**0.0007**	1.14 (0.32–18)	0.99 (0.2–3.43)	−2.14	**0.016**	0.97 (0.28–52)	0.85 (0.34–7.98)	−2.32	**0.010**
PT, s [635]	10.9 (1.43–96.5)	10.95 (9.6–58.8)	−0.45	0.326	11.1 (9.3–79)	10.9 (9.6–58.8)	−0.67	0.251	10.8 (1.43–96.5)	11 (9.9–33.3)	0.09	0.465
PTT, s [384]	32.8 (0.9–150)	31.2 (23.1–147.8)	−1.32	0.094	34.1 (20.7–150)	32.1 (23.1–147.8)	−1.25	0.105	31.3 (0.9–139)	31 (25.9–42.8)	−0.50	0.309
INR [637]	1.0 (0.37–38.4)	1 (0.46–6.2)	−0.73	0.234	1 (0.9–8)	1.0 (0.46–6.2)	−0.87	0.192	1 (0.37–38.4)	1 (0.9–3.4)	−0.24	0.406

a *P* values indicate differences between inpatients with COVID-19 without versus with PNS symptoms, and *P* less than 0.05 was considered statistically significant using the Wilcoxon Two-Sample Test.

BUN, blood urea nitrogen; CK, creatine kinase; CRP, C-reactive protein; ESR, erythrocyte sedimentation rate; INR, International normalized ratio; LDH, lactate dehydrogenase; PT, prothrombin time; PTT, partial thromboplastin time.

Bold values signify statistical significance.

In patients who had significant vertiginous symptoms (e.g. dizziness, lightheadedness, etc.) the most common treatment was oral meclizine. Additionally, in combination with metoclopramide in those with associated nausea and vomiting. Participants diagnosed with peripheral neuropathy were commonly managed with oral GABApentin. In the few patients with acute neuropathy and an associated acute kidney injury topical lidocaine provided significant symptomatic relief. No specific treatment was utilized for individuals with dysgeusia.

### Laboratory findings in patients with and without skeletal muscle injury

Table 5 represents the laboratory markers inpatients with and without skeletal muscle injury. Although an elevated CK is seen in patients with skeletal muscle injury and rhabdomyolysis, it may also be elevated in those with a myocardial injury. Patients were further stratified based on ICU admission status. The data is represented by the median value with a range. The absolute neutrophil count was lower in the total group of patients [3.6 (0.5–14.19) vs. 4.9 (0.5–83.8), *P*=0.015], and non-ICU group [2.85 (1.1–9.86) vs. 4.4 (0.5–83.8), *P*=0.011] with skeletal muscle injury. WBC count was also lower in non-ICU patients with skeletal muscle injury compared with those without such injury [4.9 (2.6–12.6) vs. 6.3 (1.3–23.4), *P*=0.045]. Elevated ferritin was observed in the total group of patients [589.5 (146–4625) vs. 378.9 (0.83–7470), *P*=0.008] and non-ICU group [581 (146–4625) vs. 272.4 (0.83–5554), *P*=0.004] with skeletal muscle injury.

**Table 5:** Laboratory findings of inpatients with COVID-19 with skeletal muscle injury

	Median (range)											
	Total (*N*=292)				ICU-Admitted (*N*=128)				Not ICU-Admitted (*N*=164)			
Characteristic [N of available/Checked data in total]	Without skeletal muscle injury (*N*=262)	With skeletal muscle injury (*N*=30)	Wilcoxon test Z score	*P* ^a^	Without skeletal muscle injury (*N*=111)	With skeletal muscle injury (*N*=17)	Wilcoxon test Z score	*P* ^a^	Without skeletal muscle injury (*N*=151)	With skeletal muscle injury (*N*=13)	Wilcoxon test Z score	*P* ^a^
Count, ×10^9^/l												
White blood cell [1220]	6.5 (1.3–23.4)	5.25 (2.6–16.7)	−1.59	0.055	7.2 (2.5–23.1)	6.1 (2.9–16.7)	−0.94	0.174	6.3 (1.3–23.4)	4.9 (2.6–12.6)	−1.69	**0.045**
Neutrophil [1190]	4.9 (0.5–83.8)	3.6 (0.5–14.19)	−2.16	**0.015**	5.5 (0.9–22.5)	4.51 (0.5–14.19)	−1.27	0.102	4.4 (0.5–83.8)	2.85 (1.1–9.86)	−2.29	**0.011**
Lymphocyte [1189]	0.9 (0.1–17.8)	0.9 (0.2–199)	0.70	0.243	0.7 (0.1–15.6)	0.9 (0.2–199)	1.03	0.150	1 (0.1–17.8)	1.15 (0.4–7.7)	0.69	0.245
Platelet [1215]	194 (6.4–598)	191 (61–572)	−1.08	0.140	177 (34–515)	192.5 (114–572)	0.03	0.488	200 (6.4–598)	175 (62–245)	−1.40	0.080
ESR, mm/h [93]	54 (2–120)	79 (67–94)	1.13	0.128	56 (2–120)	85 (76–94)	0.52	0.301	46 (7–120)	74.5 (67–82)	0.97	0.166
CRP, mg/l [797]	79.4 (0.5–2200)	105.1 (24.1–292.6)	1.47	0.070	147 (2–400)	165.15 (30.1–292.6)	0.80	0.212	52.1 (0.5–2200)	77.6 (24.1–146)	0.646	0.259
D-dimer, mg/l [659]	1.34 (0.13–5000)	1.08 (0.3–1240)	−1.21	0.112	1.55 (0.23–5000)	1.32 (0.3–1240)	−1.27	0.103	1.17 (0.13–3650)	0.89 (0.36–571)	−0.64	0.261
Fibrinogen, mg/dl [118]	509 (2–6455)	542 (294–734)	0.41	0.341	530 (2–813)	561 (478–734)	0.18	0.427	476.5 (230–6455)	418 (294–542)	−0.69	0.244
Ferritin, ng/ml [564]	378.9 (0.83–7470)	589.5 (146–4625)	2.39	**0.008**	502 (2–7470)	598 (227–2121)	−0.21	0.418	272.4 (0.83–5554)	581 (146–4625)	2.69	**0.004**
LDH, U/L [465]	295.5 (105–4706)	440 (223–986)	3.31	**0.0005**	372 (106–4706)	640 (325–986)	2.53	**0.006**	264 (105–960)	292 (223–425)	1.00	0.159
CK, U/L [292]	99 (2–60 000)	366 (201–1922)	5.62	**<0.0001**	156.5 (14–60 000)	393 (201–1922)	2.91	**0.002**	72 (3.0–159)	313 (218–846)	4.55	**<0.0001**
Aminotransferase, U/L												
Alanine (ALT) [1075]	28 (3–433)	53 (10–330)	3.44	**0.0003**	32 (3–433)	48 (10–330)	1.29	0.099	27 (3–171)	53 (28–104)	3.42	**0.0003**
Aspartate (AST) [1076]	39.5 (11–2640)	57.5 (20–359)	3.36	**0.0004**	48 (13–2640)	63 (20–359)	1.38	0.083	33 (11–223)	53 (28–104)	3.05	**0.001**
BUN, mg/dl [1204]	20 (0.9–171)	16 (6–56)	−1.59	0.056	26 (0.9–141)	16 (6–38)	−2.85	**0.002**	17 (3–171)	16 (7–56)	0.27	0.392
Creatinine, mg/dl [1211]	1 (0.42–19.07)	0.99 (0.2–7.57)	0.06	0.474	1.2 (0.42–18)	0.97 (0.2–2.16)	−1.71	0.043	0.92 (0.45–19.07)	1.17 (0.67–7.57)	1.53	0.063
PT, s [635]	11.1 (8.9–46)	10.9 (9.6–34)	−0.90	0.183	11.2 (9.3–44.8)	10.9 (9.6–11.9)	−1.56	0.060	11 (8.9–46)	11 (10–34)	0.28	0.391
PTT, s [384]	32.8 (20–147.8)	31.9 (26.8–112.7)	−0.01	0.496	33.4 (20.7–147.8)	31.7 (26.8–112.7)	−0.53	0.297	32.2 (20–83)	35 (30.4–67.2)	0.95	0.172
INR [637]	1 (0.9–4.8)	1 (0.9–3.5)	−0.17	0.431	1 (0.9–3)	1.05 (1–1.1)	−0.14	0.446	1 (0.9–4.8)	1 (0.9–3.5)	−0.20	0.422

a *P* values indicate differences between inpatients with COVID-19 without versus with skeletal muscle injury, and *P* less than 0.05 was considered statistically significant using the Wilcoxon Two-Sample Test.

BUN, blood urea nitrogen; CK, creatine kinase; CRP, C-reactive protein; ESR, erythrocyte sedimentation rate; INR, International normalized ratio; LDH, lactate dehydrogenase; PT, prothrombin time; PTT, partial thromboplastin time.

Bold values signify statistical significance.

Participants with myalgia without evidence of elevated CK were treated only supportively. Patients who had symptomatic myalgia in addition to an elevated serum CK were treated supportively with oral analgesics in addition to intravenous hydration.

## Discussion

### Demographics & comorbidities

In our study, we found that about 31% of admitted inpatients required ICU level of care for the management of COVID-19. A systematic review pooling data from 28 studies determined an ICU admission rate of 21% (95% CI, 0.12–0.34)^13^. However, variables to be taken into consideration include the dominant viral strain of SARS-CoV-2, vaccine availability, and vaccination status. Another systematic review of seven studies (1813 COVID-19 patients) found ICU patients were older (62.4 years) compared to non-ICU (46 year), and a larger proportion were men^14^. Similarly, we found that there was a significant difference between male sex and age greater than or equal to 60 years regarding the ICU admission. In a study by Grasselli *et al*.^15^ on 3988 patients with COVID-19 requiring ICU level of care, similar risk factors were found to be associated with ICU admission including hypercholesterolemia (hazard ratio, 1.25; 95% CI, 1.02–1.52), type 2 diabetes (hazard ratio, 1.18; 95% CI, 1.01–1.39), and male sex. Another meta-analysis from 2020 of 61 studies (10 000 COVID-19 cases) found that male sex, older age, and comorbidities were closely associated with disease severity and prognosis related to COVID-19^16^. The increased morbidity and mortality seen in men could be secondary infectivity mechanisms related to angiotensin-converting enzyme 2 receptor^17^.

### Systemic symptoms

Our data showed that fever was the symptom most prevalent in those requiring ICU level of care. Likewise, a prior meta-analysis pooled data from 58 studies and found that fever was observed in 51% of patients (95% CI, 45–57%; *I* ^2^=78.9%)^18^. A retrospective cohort analysis revealed that patients admitted to the ICU had higher peak temperature in comparison to those who were not [103.3 °F (IQR 1.7) vs. 100.0 °F (IQR 3.5), *P*<0.0001]^19^. It is biologically plausible that the presence of fever is indicative of a severe infection compared to a mild inflammatory response inpatients without a measurable fever.

### Laboratory markers

In our cohort we found a multitude of abnormal laboratory markers in the ICU-admitted patients including elevated BUN, creatinine, acute phase reactants, WBC count, neutrophil count, D-dimer, thrombocytopenia, and lymphopenia. Figliozzi *et al.* ^20^ pooled data in a meta-analysis of 49 studies and found that increased procalcitonin [odds ratio (OR) 4.8, CI 2.034–11.31], D-dimer (OR 3.7, CI 1.74–7.89), and thrombocytopenia (OR 6.23, CI 1.031–37.67) conveyed the highest odds for mortality. Among ICU patients, we observed an elevation in D-dimer, which has been implicated in the coagulation cascade and part of the pathogenesis of acute coronary syndrome^21^. Similarly, one of the common neurological manifestations inpatients admitted to the ICU for COVID-19 was cerebrovascular disease which may have coinciding factors of pathogenesis^22,23^. Proinflammatory markers and acute phase reactants such as CRP, ferritin, and LDH have been observed in severe COVID-19 cases^24–26^.

### Neurological manifestations

Although respiratory disease is the most common presentation of COVID-19, extrapulmonary symptoms and neurological manifestations are commonly reported^2–5^. CNS signs and symptoms were recorded in 41.5% of patients from our cohort. ICU-admitted patients had a higher prevalence of impaired consciousness (36.8 vs. 22.6%) and acute cerebrovascular disease (2.6 vs. 0.94%) in comparison to the non-ICU group. A review of 765 patients with COVID-19 revealed that 18% had neurological symptoms and disease, with dysgeusia being one of the most common^27^. Within the spectrum of CNS involvement encephalopathy has been one of the most common neurological signs reported early on during the pandemic and was seen more commonly in the ICU-admitted patients^28^. Cerebrovascular accident has an incidence of 1.5% (range: 0.1–6.9%) among patients with COVID-19, which is similar to our present data (1.45%). The wide range is attributed to the level of acuity of disease, from out-patient management to ICU admission. Patients with COVID-19 have a fivefold increased change of having a stroke (OR 5.1, 95% CI 2.72–9.54). Immune mediated thrombosis and subsequent hypercoagulability are among the underlying etiology for this neurological manifestation^3,29^.

In addition to CNS involvement, COVID-19 has been reported to affect the PNS and cause muscle skeletal injury as well^2,30^. PNS manifestations were not overall difference between ICU and non-ICU patients with COVID-19. Common PNS symptoms reported in the literature include dysnosmia and dysgeusia, peripheral neuropathy, and on the spectrum of Guillain–Barré syndrome^2,12^. Cases of myopathy and myositis have also been seen and supported by imaging findings including muscle edema^31,32^. Similar to CNS pathogenic mechanisms, PNS involvement and muscle injury have been suggested to be associated with upregulation of the immune system including elevations of cytokines such as interleukin-6 and interferon-γ^32–34^.

The presence of both CNS and PNS symptoms implies that SARS-CoV-2 may have neurotropic mechanisms of invasion into the nervous system or could exert a secondary effect on the immune system leading to neurological manifestation. Viruses can exert neurotropism through various mechanisms including blood brain barrier (BBB) breakdown, retrograde neuronal propagation, cellular mimicry amongst others^35^. Endothelial dysfunction has been recognized as an important pathophysiologic mechanism associated with COVID-19^2,3^. Invasion of endothelial cells by SARS-CoV-2 and the resulting endothelitis may be seen in multiorgan involvement and BBB breakdown observed in COVID-19^36^. Neurons and glial cells express the angiotensin-converting enzyme 2 receptor, which may serve as a mechanism of SARS-CoV-2 neuroinvasion. Systemic hyperimmune responses including the elevation of interleukin-6 and other cytokines have been observed in COVID-19 in those with neurological sequelae^37^. Interventions such as defibrotide and heparinase inhibitors have been proposed to help mitigate this^38^.

Individuals infected with neurological sequelae of COVID-19 have shown signs of BBB dysfunction and endotheliopathy including elevated albumin, protein, and immunoglobulin index in CSF. Although indirect neuroinvasion of SARS-CoV-2 has been observed, direct CSF detection has remained inconsistent. A retrospective analysis of 150 lumbar punctures in 127 patients with COVID-19 determined that direct detection of SARS-CoV-2 in CSF was rare if occurring at all^39^. Likewise, mechanisms of ‘neuro-evasion’ or the inability to directly detect the virus in the CSF has also been theorized. Such mechanisms may include trans-neuronal spread with SARS-CoV-2 rarely entering the extracellular space and thus avoiding CSF detection^40^. Further research on both mechanisms of neuroinvasion and evasion is necessary.

It has been demonstrated here that neurological signs and symptoms are observed inpatients infected with SARS-CoV-2 and are associated with severe CVOID-19 requiring ICU admission. Recognition of these symptoms can aid clinicians in critical decision making for early utilization of advanced therapeutic measures including remdesivir, dexamethasone, and baricitinib^41^.

### Study, strengths, and limitations

The neurological manifestations from COVID-19 have been reported in the past. Our research provides robust verification of neurological manifestations and comprehensive laboratory analysis. Furthermore, we stratified laboratory data based on the localization of symptoms in the CNS, PNS, and skeletal muscle. No study is without limitations. Data was collected retrospectively; therefore, there is a possibility of missed data. For instance, information on skeletal muscle injury was only available from 292 patients, as CK was only checked or available in 292 cases upon admission. The functional status including modified ranking score prior to admission was not collected. It is noteworthy that although our definition of skeletal muscle injury was based on the early study published in the first wave of COVID-19 outbreak in China^12,^ more recent studies^42,43^ have indicated that myalgia may not be associated with hyperCKemia in patients with COVID-19. Instead, it was found that COVID-19 patients with history of falls (i.e. patients admitted after falling at home) had CK levels higher than others even without clinical evidence of muscle trauma on ER reports^43^. Overall, CK is also a nonspecific enzyme for skeletal muscle and may be elevated in those with myocardial insult. Therefore, these factors need to be considered when interpreting data from skeletal muscle injury related to COVID-19. Future directions include further research on mechanisms of SARS-CoV-2 neuroinvasion and its prevention as well as the efficacy on the early use of therapeutic agents based on neurological manifestations (i.e. impaired consciousness and acute cerebrovascular disease) of severe COVID-19. Longitudinal follow-up of patients discharged from the hospital was not performed. Therefore, severity and progression of neurological sequelae cannot be determined. The neurological sequelae and laboratory biomarkers reported in the study need to be validated through further prospective studies to ensure biological plausibility.

In the present study, we also did not collect data from non-COVID patients in the time frame that data was collected for patients hospitalized due to COVID-19. Unfortunately, we do not have any statistical analysis for comparison between two groups of patients with COVID-19 and those without COVID-19 (especially those admitted for pneumonia, acute respiratory distress syndrome, or sepsis) to assess the differences between neurological manifestations especially in those admitted to the ICU. As our data is based on general/neurological clinical manifestations, laboratory markers, and neurodiagnostic evaluations upon admission, it is possible that a subgroup of patients admitted due to COVID-19 could have and/or developed superimposed other infections/conditions including pneumonia, acute respiratory distress syndrome, or sepsis not-related to COVID-19 upon or during hospitalization. Therefore, some of the neurological or other clinical manifestations could have been due to these conditions rather than COVID-19 itself. Accordingly, this issue and limitation should be considered when interpreting the results of the present study.

## Conclusions

In our comprehensive retrospective study encompassing 1248 COVID-19 patients, a noteworthy 31% required ICU care for disease management. Intriguingly, ICU-admitted patients were predominantly older (age≥60 years) and male. These individuals frequently exhibited a higher prevalence of comorbidities, such as heart failure, coronary artery disease, diabetes, and hyperlipidemia. ICU patients were also more likely to experience CNS symptoms, particularly impaired consciousness, and acute cerebrovascular disease. Systemic symptoms correlating with ICU admission included fever, and a heightened mortality rate was observed among COVID-19 patients in the ICU. An extensive range of abnormal laboratory markers was identified in ICU-admitted patients, including lymphopenia, thrombocytopenia, elevated WBC count, neutrophil count, BUN, creatinine, D-dimer, and acute phase reactants. Our study offers valuable clinical insights into COVID-19, assisting healthcare providers in enhancing their understanding of the disease’s neurological manifestations, particularly for patients admitted to the ICU.

## Ethical approval

The study was conducted in accordance with the Declaration of Helsinki and approved by the Institutional Review Board (or Ethics Committee) of University of Massachusetts Chan Medical School (protocol code H00020983, approved on 26 August 2020).

## Consent

Written informed consent was obtained from the patients for publication and any accompanying images. A copy of the written consent is available for review by the Editor-in-Chief of this journal on request. Written informed consent was obtained from the patients’ parents/legal guardians for publication and any accompanying images. A copy of the written consent is available for review by the Editor-in-Chief of this journal on request.

## Sources of funding

This research received no external funding. M.G. is supported by a clinical research training scholarship in ALS funded by The ALS Association and The American Brain Foundation, in collaboration with the American Academy of Neurology as well as NIH-funded Wellstone fellowship training grant (NIH 5P50HD060848-15) for research on FSHD.

## Author contribution

M.G.: outlined the performed rigorous literature search, conceived, and designed the study, supervised the study team and data collection, performed the formal statistical analysis, prepared the tables, and wrote the original draft of the manuscript; B.S.S. and M.A.G.: contributed to the formal statistical analysis and wrote the original draft of the manuscript; P.P., J.Q., S.S., J.B., A.P., S.C., D.T.S., E.M., N.T.H., A.B., and V.L.: performed rigorous clinical chart review from patients’ electronic medical records, collected the data, and edited the manuscript. All authors have read and agreed to the published version of the manuscript.

## Conflicts of interest disclosure

The authors declare that they have no financial conflict of interest with regard to the content of this report.

## Research registration unique identifying number (UIN)

ResearchRegistry.com https://www.researchregistry.com/browse-theregistry#home/registrationdetails/6478023642fa5b0028aacf19/. UIN: researchregistry9101.

## Guarantor

Mehdi Ghasemi.
